# Japanese pediatric patient with refractory steroid-resistant ulcerative colitis successfully treated with Tofacitinib: A case report

**DOI:** 10.1097/MD.0000000000031757

**Published:** 2022-11-11

**Authors:** Toshihiko Kakiuchi, Masato Yoshiura

**Affiliations:** a Department of Pediatrics, Faculty of Medicine, Saga University, Saga, Japan.

**Keywords:** child, Japanese, tofacitinib, ulcerative colitis

## Abstract

Tofacitinib is an orally administered selective Janus kinase inhibitor. Its efficacy and safety in adults with moderately to severely active ulcerative colitis (UC) have been evaluated in clinical trials; however, its efficacy in pediatric patients with UC is limited.

**Patient concerns:**

A 9-years-old boy was referred to our hospital with chief complaints of diarrhea and bloody stool for 2 months. Colonoscopy revealed total colitis-type UC. His pediatric UC activity index score was 40, indicating moderately active UC.

**Diagnosis:**

UC.

**Interventions:**

Vedolizumab, golimumab, and ustekinumab were introduced because of steroid-resistant refractory UC; however, none of these biologics were effective or the effect was short-lived. Therefore, tofacitinib was administered 5 mg twice a day.

**Outcomes:**

The patient achieved UC remission after tofacitinib treatment, leading to maintained remission without adverse events.

**Lessons:**

To the best of our knowledge, this is the first pediatric case of moderately active UC that was successfully treated with tofacitinib in Japan. Tofacitinib is a safe drug for pediatric patients with moderately active UC. Even in steroid-dependent cases refractory to other biologics, tofacitinib can result in remission induction and maintenance effects. In children and adults, high-dose tofacitinib during induction therapy may be unnecessary to reduce adverse events.

## 1. Introduction

The Janus kinase (JAK) family of nonreceptor protein-tyrosine kinases consists of JAK1, JAK2, JAK3, and tyrosine kinase 2.^[1]^ Tofacitinib and upadacitinib are JAK antagonists that are used for the treatment of rheumatoid arthritis and ulcerative colitis (UC).^[2]^ Tofacitinib is an orally administered selective JAK inhibitor and is a small molecule that selectively targets JAK1 and JAK3 in the JAK-signal transduction and activator of transcription pathway, thereby downregulating the activity of proinflammatory cytokines.^[3]^ The efficacy and safety of tofacitinib in adults with moderately to severely active UC has been evaluated in clinical trials, including a dose-ranging phase 2 induction trial,^[3]^ 2 phase 3 induction trials (OCTAVE Induction 1 and 2),^[4]^ a 52-weeks phase 3 maintenance trial (OCTAVE Sustain),^[4]^ and an ongoing, open-label, long-term extension trial (OCTAVE Open).^[5]^ However, the efficacy of tofacitinib in pediatric patients with UC is limited.^[6]^

In March 2013, tofacitinib was approved in Japan as a new treatment drug for rheumatoid arthritis in adults and permitted for use in adults with UC in May 2018. To our knowledge, there are no reports of Japanese children with UC who received long-term tofacitinib therapy. Herein, we report a Japanese pediatric case of refractory steroid-resistant UC that was successfully treated with tofacitinib.

## 2. Case report

A 9-years-old boy was referred to our hospital with chief complaints of diarrhea and bloody stool for 2 months. At a pediatric clinic, he was diagnosed with acute infectious gastroenteritis and prescribed fosfomycin (50 mg/kg) and antiflatulents for 7 days; however, his symptoms did not improve. He was referred to our hospital because of anorexia and weight loss. On admission, his physical examination revealed the following: height, 135.5 cm (standard deviation + 0.1); bodyweight, 30.8 kg (standard deviation − 0.2), 2.1 kg below weight 2 months ago; body temperature, 36.3°C; heart rate, 78 beats per minute; and blood pressure, 94/50 mm Hg. He had increased bowel sounds. His blood test results were as follows: white blood cell count, 17,500 (normal range [NR]: 7000–15,000) cells/mL; hemoglobin, 9.4 (NR: 13.7–16.8) g/dL; and platelet count, 847 × 103 (NR: 158–348 × 103) cells/mL. His laboratory test findings were as follows: total protein, 6.5 (NR: 6.8–8.1) g/dL; albumin, 3.1 (NR: 4.1–5.1) g/dL; aspartic aminotransferase, 25 (NR: 20–45) IU/L; alanine aminotransferase, 24 (NR: 4–24) IU/L; blood urea nitrogen, 8.5 (NR: 8–20) mg/dL; sodium, 143 (NR: 137–147) mEq/L; potassium, 4.8 (NR: 3.6–5.2) mEq/L; C-reactive protein, 0.25 (NR: <0.14) mg/dL; serum amyloid A protein, 251.7 (NR: <8.0) µg/mL; erythrocyte sedimentation rate, 50 (NR: <17) mm/H; bicarbonate, 19.1 (NR: 21.0–27.0) mmoL/L; lactic acid, 21 (NR: 4–16) mg/dL; and immunoglobulin G, 954 (NR: 357–989) mg/dL. No infection was detected in the stool culture tests, and the fecal calprotectin level was 15,700 (NR: <50) mg/kg. Stool adenovirus, rotavirus, and norovirus antigen tests were negative.

**Figure 1.**
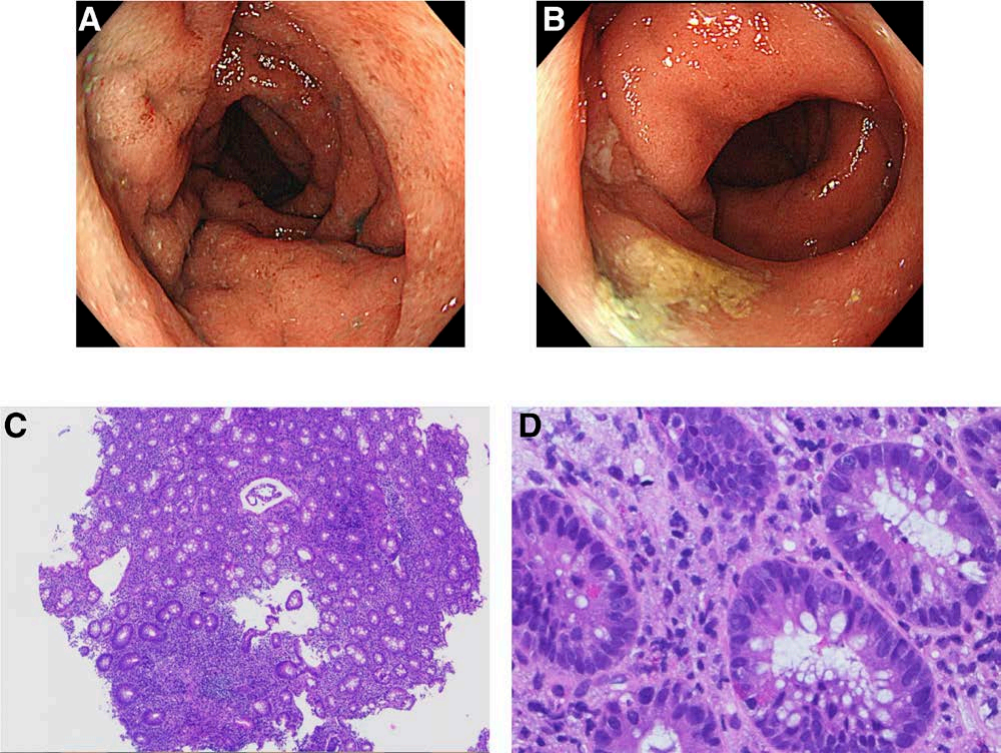
Endoscopic images and pathological findings during diagnosis. The mucous membrane was reddish and bled easily. Edema was observed from the distal side of the ascending colon to the rectum (A, sigmoid colon; B, rectum). The Ulcerative Colitis Endoscopic Index of Severity score was 2. On pathological examination, erosion, degeneration, and regeneration of the epithelium were found in the mucosa. Edema, hyperemia, and infiltration of inflammatory cells (lymphocytes, plasma cells, and neutrophils) were observed in the lamina propria (C and D). Many cryptitis and crypt abscesses were noted.

His symptoms were indicative of UC; therefore, we performed a total colonoscopy (TCS) under sedation with thiamylal sodium. Figure 1A and B presents the TCS findings. The mucous membrane was reddish and bled easily. Edema was observed from the distal side of the ascending colon to the rectum. On pathological examination, erosion, degeneration, and regeneration of the epithelium were found in the mucosa. Edema, hyperemia, and infiltration of inflammatory cells (lymphocytes, plasma cells, and neutrophils) were observed in the lamina propria. Many cryptitis and crypt abscesses were observed (Fig. 1C and D). Given these findings, the patient was diagnosed with UC. His Ulcerative Colitis Endoscopic Index of Severity (UCEIS)^[7]^ score was 2 and the pediatric ulcerative colitis activity index (PUCAI)^[8]^ score was 40.

**Figure 2.**
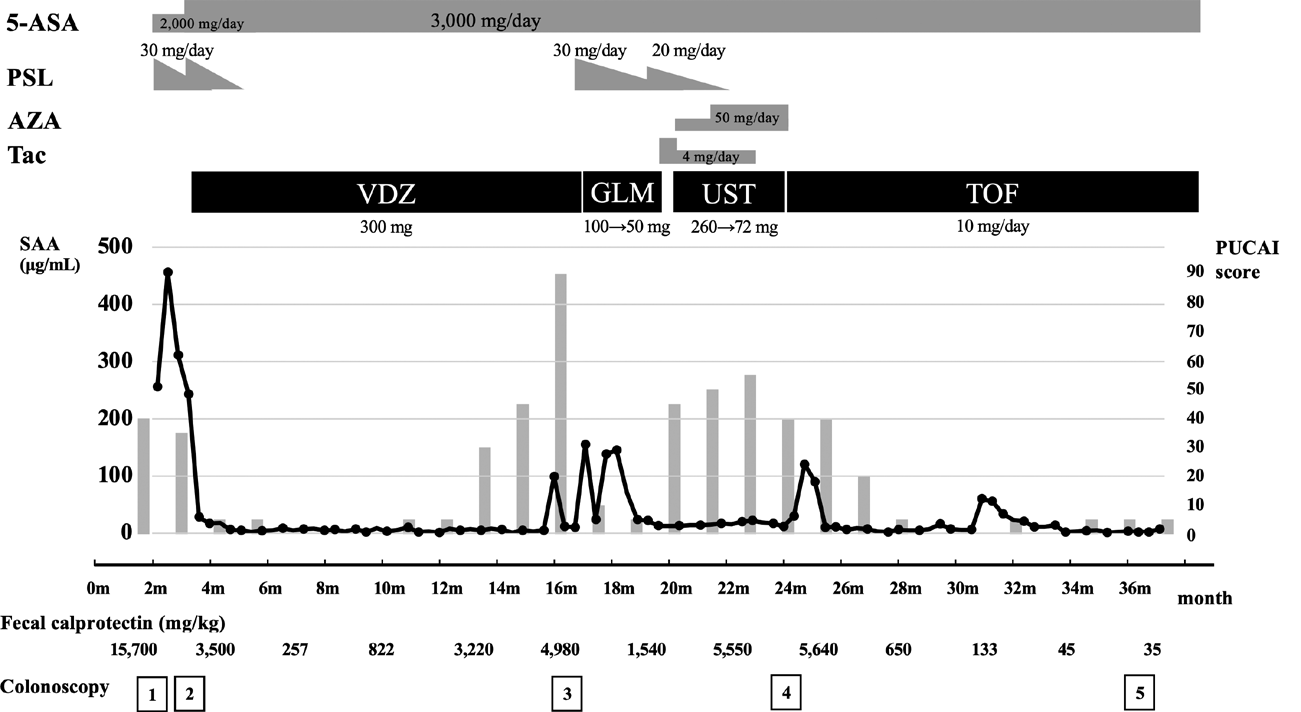
Patient’s clinical course and treatment for ulcerative colitis. Vedolizumab, golimumab, and ustekinumab were introduced because of steroid-resistant refractory UC; however, none of the biologics were effective or the effect was short-lived. Therefore, tofacitinib was administered 5 mg twice a day. Consequently, the patient was in clinical remission 16 weeks after starting tofacitinib. He remained in clinical remission without relapse at 52 weeks of tofacitinib and had maintained endoscopic remission on his fifth colonoscopy. No obvious adverse events were observed during tofacitinib use. Bar graph indicated PUCAI score. 5-ASA = 5-aminosalicylate acid, AZA = azathioprine, GLM = golimumab, PSL = prednisolone, PUCAI = pediatric ulcerative colitis index, SAA = serum amyloid A, Tac = tacrolimus, TOF = tofacitinib, UST = ustekinumab, VDZ = vedolizumab.

**Figure 3.**
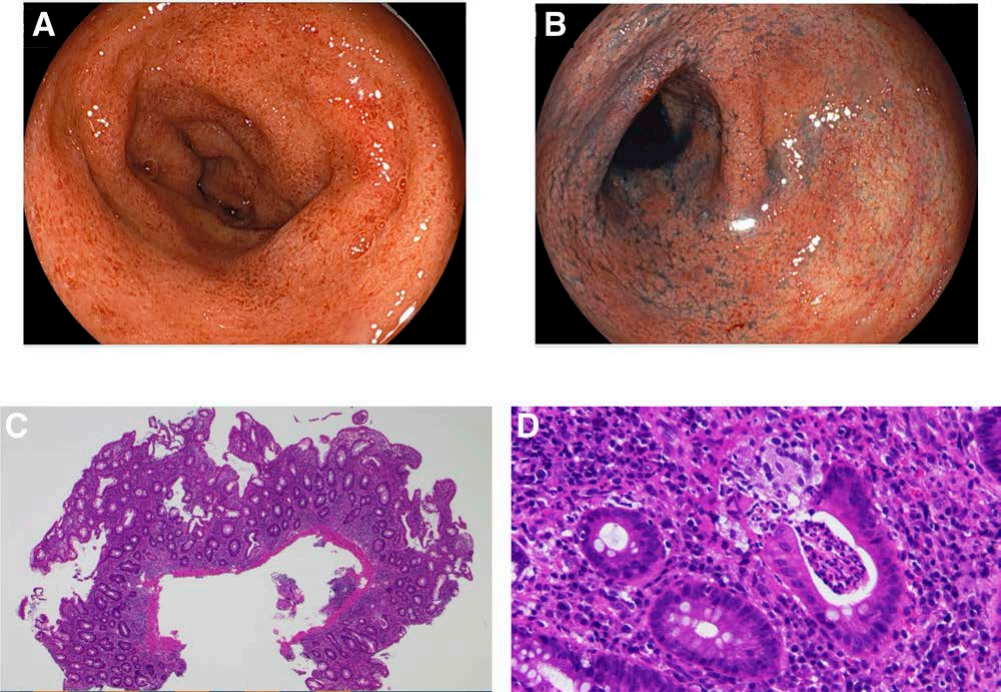
Sigmoidoscopy images and pathological findings before tofacitinib administration. The sigmoidoscopy revealed coarse, granular, and edematous mucosa with erosion (A and B: sigmoid colon). The Ulcerative Colitis Endoscopic Index of Severity score was 4. On pathological examination, diffuse chronic inflammatory cell infiltration, goblet cell depletion, cryptitis, and crypt abscesses were observed, which consistent with active ulcerative colitis (C and D).

As shown in Figure 2, he was initially treated with 5-aminosalicylate acid and 1 mg/kg prednisolone, which did not improve his symptoms. The second TCS was performed 1 month later, which revealed coarse, granular, edematous mucosa from the cecum to the rectum without erosion or ulcer (UCEIS score 3). Given the absence of therapeutic response to increased doses of 5-aminosalicylate acid and prednisolone, 300 mg vedolizumab was introduced as a biologic agent for refractory steroid-resistant UC with pancolitis type. After the vedolizumab treatment, clinical remission was successfully induced quickly, and steroid-free clinical remission was maintained for approximately 11 months thereafter, but the gastrointestinal symptoms gradually recurred and worsened to PUCAI score 90. The third TCS revealed coarse, granular, edematous mucosa from the descending colon to the rectum with erosion (UCEIS score 4). The resumption of 1 mg/kg prednisolone and conversion of vedolizumab to golimumab (100 mg for the first time and 50 mg thereafter) improved his symptoms rapidly (PUCAI score 10); however, his PUCAI score relapsed quickly with prednisolone dose reduction. Since the clinical symptoms did not respond to increased prednisolone dose, tacrolimus (target trough level 10–15 ng/mL) was introduced for remission induction. Two weeks later, ustekinumab (260 mg subcutaneous injection for the first time, followed by 72 mg by drip every 8 weeks) was replaced with golimumab with tacrolimus dose reduction (target trough level 5–10 ng/mL), and azathioprine (50 mg/day) was started simultaneously. Since ustekinumab was administered 3 times at 8-weeks intervals and no effect was observed, sigmoidoscopy was performed, which revealed coarse, granular, edematous mucosa with erosion (UCEIS score 4) (Fig. 3A and B). On pathological examination, diffuse chronic inflammatory cell infiltration, goblet cell depletion, cryptitis, and crypt abscesses were observed, which were consistent with active UC (Fig. 3C and D).

Tofacitinib was introduced after obtaining approval from the Ethics Committee of Saga University Hospital (Approval date: January 4, 2021). The patient and her family were offered tofacitinib as a potential salvage therapy and were informed that the medication is not licensed yet for children with UC, but it is licensed for adults with UC and children with juvenile idiopathic arthritis overseas. They agreed to try tofacitinib with informed consent, understanding its benefits, risks, potential complications, and side effects. After identifying that he was positive for varicella-zoster virus antibody, tofacitinib was introduced at 5 mg twice a day, and the policy was to continue at the same dose from the 8th weeks onwards. As a result, he was in clinical remission (PUCAI score 5) 16 weeks after starting tofacitinib. He remained in clinical remission without relapse at 52 weeks of tofacitinib and had maintained endoscopic remission on his fifth TCS. No remarkable adverse events (AEs) were observed during tofacitinib use.

## 3. Discussion

The disease course of this case provides 2 important indications. First, tofacitinib was effective for a pediatric patient with moderately active UC. Second, the low induction dose of tofacitinib was found to be effective and safe for a pediatric patient with UC.

Tofacitinib has shown efficacy to moderate-to-severe UC in adults. This finding was clear in OCTAVE trials 1 and 2, where tofacitinib was given to patients with moderate-to-severe UC who failed at least 1 class of medical therapy.^[4]^ High-certainty evidence suggests that tofacitinib is superior to placebo for the induction of clinical and endoscopic remission at 52 weeks in participants with moderate-to-severe UC who had a clinical response after 8 weeks of induction treatment with tofacitinib (10 mg twice daily) or placebo.^[9]^ The American Gastroenterology Association recommended tofacitinib as second- or third-line therapy for those who failed at least 1 class of biologic therapy.^[10]^ On the contrary, data on tofacitinib use in pediatric inflammatory bowel disease are limited.^[11]^ Despite the limited evidence on the use of tofacitinib in children with UC, some case series reported similar outcomes. The first study involved 5 childrens with severe refractory UC who received tofacitinib at a dose of 10 mg twice daily over a mean observation period of 9.7 weeks. This study showed that high-dose tofacitinib resulted in clinical response and/or steroid-free remission in all patients with UC.^[11]^ Arajmi et al highlighted the safety and efficacy of tofacitinib in a 13-years-old girl with severe refractory UC.^[12]^ To our knowledge, this is the first report of the use of tofacitinib in a pediatric patient with UC in Asia. In a cohort study of Hillary et al, tofacitinib induced rapid clinical response with sustained efficacy in nearly half of their patients. This study provided encouraging evidence on the efficacy and safety of tofacitinib as part of the treatment paradigm for young patients with moderate-to-severe inflammatory bowel disease.^[6]^ The present case showed its efficacy on a Japanese pediatric patient with refractory steroid-resistant UC, which was refractory to 3 biologics, namely, vedolizumab, golimumab, and ustekinumab. UC morbidity differs with race,^[13]^ and 1 of the possible reasons is the effect of UC-related genes and human leukocyte antigen. In a large-scale genome-wide association study from Europe and North America, approximately 50 UC-related gene regions have been revealed, but the results were different from them in Japan.^[14,15]^ Similarly, the effects and safety of therapeutic agents, including biologics, on UC may also vary by race. As data on Japanese children are scarce, data accumulation for this patient population is necessary hereon.

The safety of tofacitinib for the treatment of UC in adult patients has been evaluated in a phase 2 trial,^[3]^ 3 large phase 3 clinical trials,^[4]^ and an ongoing, open-label, long-term extension trial.^[16–19]^ In addition to demonstrating the efficacy of tofacitinib as an induction therapy, OCTAVE 1 and 2, the 2 phase 3 induction trials, also showed a higher overall infection and serious infection rate in the tofacitinib group than in the placebo group. In the phase 3 maintenance trial of tofacitinib (OCTAVE Sustain), the treatment group had higher rates of any infection and reactivation of herpes zoster than the placebo group. Information on the safety of tofacitinib in pediatric patients with UC is limited, but Hillary et al reported that most of their patients were on 10 mg BID most of the time and had no cases of thrombosis, clinically significant hyperlipidemia, or other cardiovascular or oncological AEs. Notable AEs attributed to tofacitinib in the adult trials were increased risk of cardiovascular events, infection, and lipid elevations.^[4,20]^ Recent drug safety communications from the FDA have reported risks of pulmonary embolism and death in adult patients with comorbidities receiving higher doses of tofacitinib.^[21]^ Deepak et al reported that the risk of herpes zoster reactivation and venous thromboembolism increased dose-dependently, and dose de-escalation of tofacitinib must be performed to the lowest clinically feasible dose. To reduce AEs, tofacitinib administration at a high dose during the induction period may not be necessary in children, and a maintenance dose may be introduced from the beginning. Essentially, we safely treated our pediatric patient with UC, without high-dose tofacitinib during the induction period.

In conclusion, tofacitinib is a safe drug for pediatric patients with moderately active UC, and even in steroid-dependent cases refractory to other biologics, tofacitinib can be result in remission induction and maintenance effects. Additionally, in children and adults, high-dose tofacitinib during induction therapy may be unnecessary to reduce AEs.

## Acknowledgements

We would like to thank the outpatient nurses and medical support staff of our hospital. The authors thank the patient’s family for providing consent and granting permission to drafting and publication of this case report.

## Author contributions

**Conceptualization**: Toshihiko Kakiuchi.

**Date curation**: Toshihiko Kakiuchi and Masato Yoshiura.

**Formal analysis**: Toshihiko Kakiuchi.

**Investigation**: Toshihiko Kakiuchi and Masato Yoshiura.

**Methodology**: Toshihiko Kakiuchi and Masato Yoshiura.

**Project administrations**: Toshihiko Kakiuchi.

**Supervision**: Toshihiko Kakiuchi.

**Validation**: Toshihiko Kakiuchi.

**Writing – original draft**: Toshihiko Kakiuchi.

**Writing – reviewing & editing:** Masato Yoshiura.
